# Neurosurgical scalp incisions should preserve at least one vascular axis: Two case reports

**DOI:** 10.1016/j.jpra.2026.10.007

**Published:** 2026-10-23

**Authors:** Elise Lupon, Benjamin Hertzog, Pharel Njessi, Olivier Camuzard

**Affiliations:** a Institut Universitaire Locomoteur et du Sport, Pasteur 2 Hospital, University Côte d’Azur, Nice, France; b Université Côte d'Azur, CNRS, LP2M, France

**Keywords:** Scalp reconstruction, Scalp incision, Vascular anatomy, Free flap, Latissimus dorsi, Neurosurgery, Hair transplantation, Case report

## Abstract

**Background:**

Scalp necrosis after neurosurgery is usually attributed to repeated surgery. We report two cases in which necrosis was precipitated by a single reoperation incision that disregarded scalp arterial anatomy and the previous scars, and describe how this dictated the reconstruction.

**Cases:**

Case 1: a 56-year-old woman underwent emergency decompressive hemicraniectomy two days after meningioma resection, through a circumferential incision along the bone-flap margin that islanded the cutaneous flap from every axial pedicle; the whole hemiscalp necrosed with brain exposure. Case 2: a 15-year-old boy, operated on repeatedly for subdural empyema, underwent cranioplasty through an incision that transected the superficial temporal pedicle, the last axis supplying a scalp segment already isolated by previous incisions. In both cases the extent of necrosis and the absence of any remaining axial pedicle precluded local, regional or expanded flaps; a free latissimus dorsi muscle flap anastomosed to the superficial temporal vessels was the only option. Hair restoration in Case 2 was completed by follicular unit extraction onto the flap.

**Conclusions:**

A scalp flap survives only if at least one axial pedicle enters its base. Neurosurgical incisions, particularly at reoperation, should be drawn on the arterial map and previous scars; plastic and neurosurgical teams should share this rule.

## Introduction

Scalp wound complications following neurosurgical procedures are uncommon but potentially life-threatening.^1^ Each hemiscalp receives four axial pedicles (supratrochlear/supraorbital, superficial temporal, posterior auricular and occipital; Fig. 1A), so richly interconnected that the whole scalp can survive on a single one of them.^2^ Conversely, a scalp flap survives only if at least one axis enters its base: a circumferential incision, or one interrupting the last remaining axis, converts the flap into a non-vascularised graft.

**Fig. 1:**
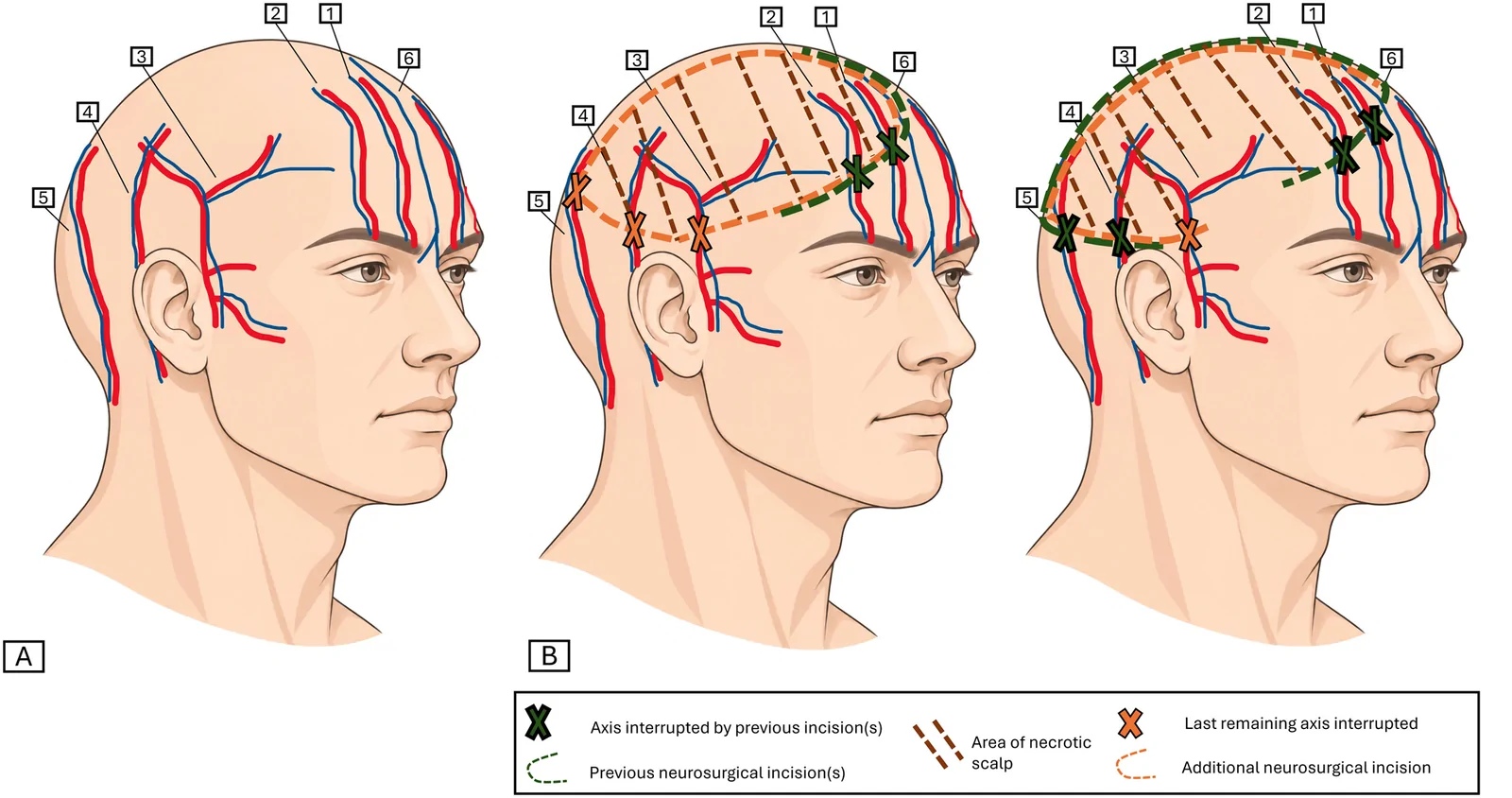
Scalp vascular axes and incisions in the two cases (right lateral view). (A) Normal anatomy of the hemiscalp: 1, supratrochlear vessels; 2, supraorbital vessels; 3, superficial temporal vessels; 4, posterior auricular vessels; 5, occipital vessels; 6, median frontal vein. (B) (Left) Case 1: the frontotemporal pterional incision of the tumour resection (green dashed line) had already interrupted the supratrochlear (1) and supraorbital (2) supply (green crosses). Two days later, the decompressive craniectomy incision (orange dashed line) reopened it and extended it temporo-parietally along the whole margin of the enlarged bone flap, interrupting the superficial temporal (3), posterior auricular (4) and occipital (5) supply (orange crosses) and islanding the flap; the whole hatched area necrosed. (Right) Case 2: the previous incisions (green dashed lines) had already interrupted the occipital (5), posterior auricular (4), supraorbital (2) and supratrochlear (1) supply (green crosses), leaving the superficial temporal pedicle (3) as the only remaining axis; the cranioplasty incision (orange dashed line), which reopened the periauricular incisions and extended them by a few centimetres, transected this last pedicle (orange cross). The hatched area necrosed; the narrow strip of scalp outside the incised area survived on random anastomotic supply. *Printed with permission; copyrights retained by Elise Lupon, MD, PhD.*

Although the free latissimus dorsi (LD) flap is well established for extensive scalp defects,^3^^,^^4^ the need for it is frequently created upstream by the incisions of previous procedures.^5^ We report two cases in which one such incision caused total scalp-flap necrosis and left free tissue transfer as the only option.

## Case 1

A 56-year-old woman underwent preoperative embolisation and partial resection (Simpson grade IV) of a right sphenoid wing meningioma (WHO grade 2) through a frontotemporal pterional incision. Two days later, a malignant right middle cerebral artery infarction required emergency decompressive fronto-parieto-temporal craniectomy: the frontotemporal incision, which had already interrupted the supraorbital and supratrochlear supply, was reopened and extended temporo-parietally along the whole margin of the enlarged bone flap, interrupting the superficial temporal, posterior auricular and occipital supply, so that the cutaneous flap was islanded exactly like the bone flap (Fig. 1B, Left). Within days the entire hemiscalp flap necrosed, exposing the brain, with a cerebrospinal fluid (CSF) leak (Fig. 2, Left).

**Fig. 2:**
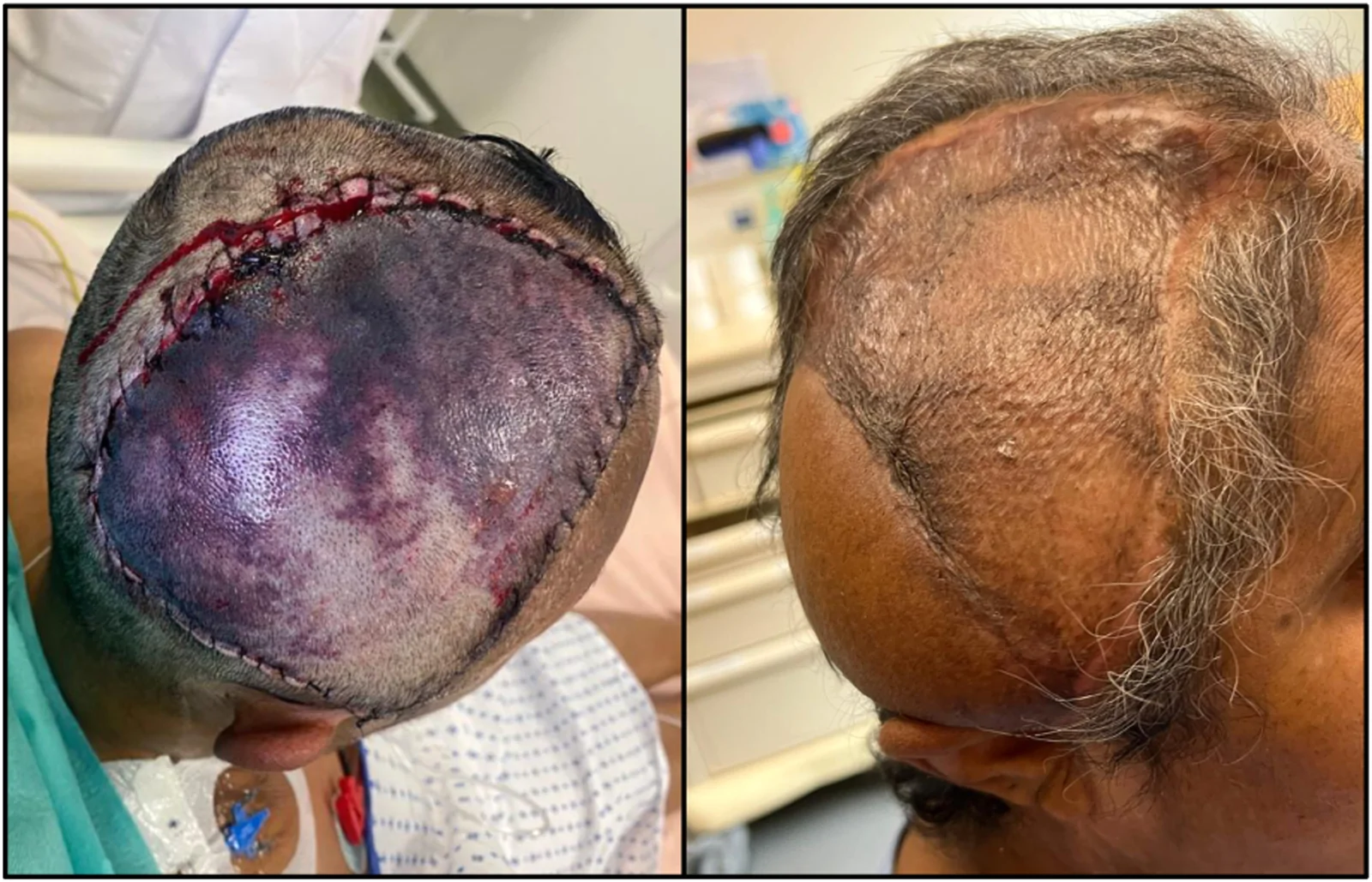
Case 1. (Left) On referral, 5 days after decompressive hemicraniectomy: total necrosis of the hemiscalp flap within the circumferential incision, with exposed brain. (Right) Result at 5 months, after debulking of the paddle and replacement of the cryopreserved autologous bone flap. *Printed with permission; copyrights retained by the operator, Elise Lupon, MD, PhD.*

After debridement, the defect involved most of the hemiscalp (approximately 20 × 15 cm), with exposed dura and brain and no bone (Supplemental Table 1). The residual scalp, crossed by both incisions, retained no axial pedicle: no local, regional or expanded flap was possible, and the exposed brain precluded delay. Five days after craniectomy, a free LD muscle flap (approximately 22 × 15 cm) with a monitoring skin paddle was harvested. The superficial temporal vessels, dissected at the pre-auricular level proximal to the scars, were patent with good Doppler flow; end-to-side anastomoses were performed to the artery and its venae comitantes. The muscle covered the whole defect (Supplemental Figure 1); a split-thickness skin graft was applied on postoperative day 5, with closure of the CSF fistula. The course was uneventful; the paddle was debulked at 3 and 4 months, and the cryopreserved autologous bone flap was replaced at 5 months (Fig. 2, Right).

## Case 2

A 15-year-old boy underwent emergency frontoparietal craniotomy for pansinusitis complicated by subdural empyema; the bone flap was removed, and further washouts followed through additional incisions. Cranioplasty with the stored bone flap was then performed by reopening the periauricular incisions and extending them by a few centimetres, which transected the superficial temporal pedicle, the only axis still supplying the frontoparietal scalp after the previous incisions (Fig. 1B, Right). Most of this segment necrosed early, exposing the replaced bone flap; only a narrow strip outside the incised area survived on random supply (Fig. 3, Left).

**Fig. 3:**
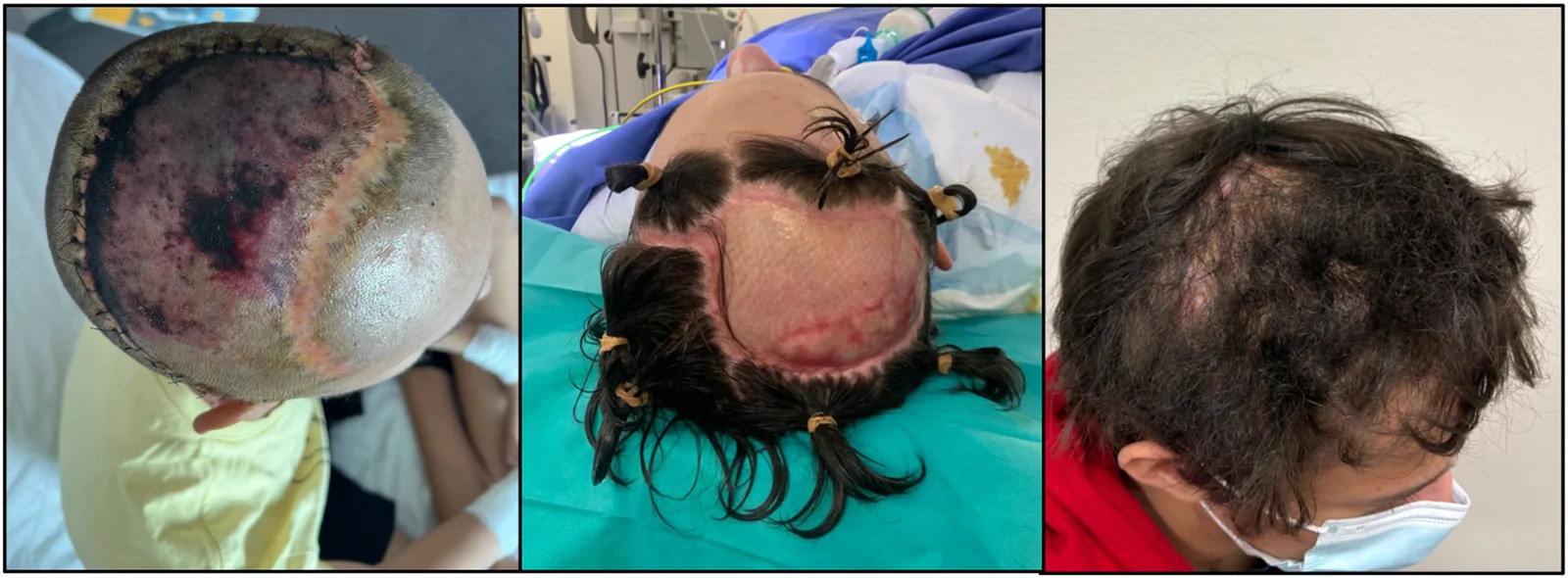
Case 2. (Left) Before reconstruction: full-thickness necrosis of the frontoparietal scalp segment with exposure of the replaced bone flap, about two weeks after cranioplasty. (Middle) At 6 months, after skin grafting and paddle excision, before placement of two tissue expanders (subsequently removed for infection complicated by septic shock and by removal of the osteolytic bone flap). (Right) At 2.5 years, 6 months after follicular unit extraction (1500 grafts) onto the flap, showing satisfactory hair density. *Printed with permission; copyrights retained by the operator, Elise Lupon, MD, PhD.*

The full-thickness defect measured approximately 15 × 12 cm over contaminated bone (Supplemental Table 1); the surrounding scalp was multiply scarred and the field infected, contraindicating local flaps and expansion. A free LD muscle flap (muscle approximately 20 × 13 cm; skin paddle 5 × 15 cm) was anastomosed end-to-end to the proximal stump of the superficial temporal artery and vein (Supplemental Figure 2). A split-thickness skin graft was applied on day 6; the paddle was later excised and its defatted skin recycled as a full-thickness graft.

Six months later, two tissue expanders were placed to restore hair-bearing scalp. Three weeks post-implantation, fever, hypotension and systemic inflammation led to their removal; despite limited local contamination, septic shock required vasopressors and intensive care. Re-exploration revealed extensive osteolysis of the bone flap, which was removed (polymicrobial cultures; antibiotics adjusted) (Fig. 3, Middle).

This disproportion between local findings and systemic response prompted an immunological work-up: lymphocyte subsets, leukocyte adhesion molecules, neutrophil oxidative burst and anti-pneumococcal antibodies were normal, and genetic testing identified a heterozygous *GFI1* variant, c.1243C>T (p.Arg415Trp), classified as of uncertain significance and also carried by the asymptomatic, non-neutropenic mother. Long-term haematological follow-up was instituted, with early antibiotic treatment of any severe febrile episode.

Two years after reconstruction, hair restoration was performed on the flap by follicular unit extraction (1500 grafts at 30–35 units/cm²) after five platelet-rich plasma sessions, with satisfactory density at 2.5 years (Fig. 3, Right).

## Discussion

Both cases could be filed under “complications of repeated scalp surgery”; we believe this is the wrong lesson. Repeated surgery erodes the vascular reserve of the scalp,^5^ but in each case necrosis was precipitated by one identifiable reoperation incision, placed without reference to the previous scars: a circumferential incision that islanded the flap (Case 1), and an incision that interrupted the last axial supply of a segment already isolated by earlier incisions (Case 2) (Fig. 1B). Whatever the neurosurgical priorities, an incision should leave at least one axial pedicle, preferably the superficial temporal or occipital, attached to every scalp segment, and at reoperation the new incision should be drawn on a map of previous scars and remaining axes, not on the outline of the bone flap.^2^

The same incisions dictated the reconstruction. Local and regional scalp flaps require an intact axial pedicle and undisturbed galea in the donor scalp, pedicled trunk muscle flaps (trapezius, latissimus dorsi) do not reach the vertex, and tissue expansion requires time and a clean field;^2^, ^6^ none was available: the defect covered most of the hemiscalp (Case 1) or a large infected frontoparietal area (Case 2), the residual scalp was multiply scarred, and brain or contaminated bone was exposed. The LD flap was selected because its broad muscle surface can reliably cover an entire hemiscalp, provides well-vascularised tissue for an infected or exposed-brain field, and has a long pedicle that reaches the superficial temporal vessels; its safety in children is established.^3^^,^^4^^,^^7^ The incisions even dictated the recipient vessels: the superficial temporal artery was found proximally at the pre-auricular level (Case 1) or used as a divided stump (Case 2), the occipital vessels having been crossed by previous incisions.

Tissue expansion for secondary hair restoration carries a substantial infection risk in previously infected or immunocompromised patients;^6^ in Case 2 it precipitated septic shock, although the subsequent immunological work-up found no confirmed deficiency. Hair transplantation directly onto the free flap, with or without platelet-rich plasma, proved a lower-risk alternative.^8^, ^9^, ^10^

Finally, the purpose of this report is not to assign blame: both incisions were made under pressure, for a malignant infarction or in an infected field, when the bone-flap outline naturally dominates planning. In our centre, reoperative scalp incisions are now discussed jointly with our neurosurgical colleagues and taught together.

## Conclusion

A scalp flap survives only if at least one axial pedicle enters its base. A circumferential incision, or one interrupting the last remaining axis of a multiply operated scalp, causes total necrosis and leaves free tissue transfer as the sole option. Neurosurgical incisions should be designed on the arterial map, a rule plastic surgeons should share with their neurosurgical colleagues.

## Patient consent

Written informed consent was obtained from both patients and from the parent/legal guardian of the minor patient for publication of their clinical data and images.

## Ethics approval

The requirement for ethics approval was waived by the institutional ethics committee for this case report.

The work has been reported in line with the 2013 CARE case report guidelines (see Supplementary Material for complete checklist).

## Financial disclosure statement

We have no financial disclosures.

## Conflict of interest

We have no conflict of interest to disclose.
