# Supplementary material for: Neurosurgical scalp incisions should preserve at least one vascular axis: Two case reports
Source: JPRAS Open. 2026 Oct 23;53:37–41. doi: 10.1016/j.jpra.2026.10.007 (PMC13742264; doi:10.1016/j.jpra.2026.10.007)
Supplement: Supplementary file 1 [file mmc1.docx]

**Supplemental Table 1. Summary of the two cases: incisions, defects, reconstruction and follow-up.**

|  | **Case 1** | **Case 2** |
| --- | --- | --- |
| **Age / sex** | 56 years / female | 15 years / male |
| **Neurosurgical history** | Right sphenoid wing meningioma (WHO grade 2): preoperative embolisation, partial resection (Simpson grade IV) through a frontotemporal pterional incision; malignant right middle cerebral artery infarction at day 1 → decompressive fronto-parieto-temporal craniectomy at day 2 (bone flap cryopreserved) | Pansinusitis with subdural empyema: emergency frontoparietal craniotomy, bone flap removed; further washouts through additional incisions; cranioplasty with stored bone flap |
| **Incision responsible for necrosis** | Previous frontotemporal incision reopened and extended temporo-parietally along the whole margin of the enlarged bone flap: cutaneous flap islanded like the bone flap | Cranioplasty incision crossing the superficial temporal territory in a scalp already divided by previous incisions |
| **Axial supply interrupted** | Supraorbital/supratrochlear by the previous pterional incision; superficial temporal, posterior auricular and occipital by the craniectomy extension (no axis left) | Superficial temporal (transected), last remaining axis of the frontoparietal segment |
| **Defect after debridement** | Near-total hemiscalp, approx. 20 × 15 cm; exposed dura and brain, no bone; CSF leak | Frontoparietal, approx. 15 × 12 cm; exposed, contaminated replaced bone flap; infected field |
| **Why no local / regional flap or expansion** | No axial pedicle left in residual scalp; scars crossing donor scalp; defect > half of scalp; exposed brain, no delay possible | No axial pedicle left; multiply scarred donor scalp; infected field (expansion contraindicated) |
| **Delay to free flap** | 5 days after hemicraniectomy | About 2 weeks after cranioplasty (demarcated necrosis) |
| **Flap** | Free LD muscle flap, approx. 22 × 15 cm, with 5 × 12 cm monitoring skin paddle | Free LD muscle flap, approx. 20 × 13 cm (ex vivo), with 5 × 15 cm monitoring skin paddle |
| **Recipient vessels / anastomosis** | Superficial temporal artery and venae comitantes, dissected at the pre-auricular level proximal to the scars; end-to-side | Superficial temporal artery and vein (proximal stump); end-to-end |
| **Skin graft on muscle** | Split-thickness, day 5 (+ closure of CSF fistula) | Split-thickness, day 6; paddle later excised and recycled as full-thickness graft |
| **Secondary procedures** | Paddle debulking at 3 and 4 months; replacement of the cryopreserved autologous bone flap at 5 months | Two tissue expanders at 6 months → infection at 3 weeks, removal, septic shock (ICU, vasopressors), removal of osteolytic bone flap; FUE 1,500 grafts + 5 PRP sessions at 2 years |
| **Relevant comorbidity** | none | Immunological work-up normal; heterozygous GFI1 variant c.1243C>T (p.Arg415Trp) of uncertain significance, also carried by the non-neutropenic mother |
| **Follow-up** | 2.5 years (Figure 2C: 5 months) | 2.5 years (Figure 3D) |

*CSF, cerebrospinal fluid; FUE, follicular unit extraction; ICU, intensive care unit; LD, latissimus dorsi; PRP, platelet-rich plasma.*

**Supplemental Figure 1.** Case 1. Free latissimus dorsi muscle flap inset with its monitoring skin paddle, before split-thickness skin grafting; the pedicle runs to the superficial temporal vessels in the pre-auricular region. *Printed with permission; copyrights retained by Elise Lupon, MD, PhD.*

**Supplemental Figure 2.** Case 2. (Left) End-to-end anastomosis of the thoracodorsal pedicle to the superficial temporal vessels through a right preauricular approach. (Right) Immediately after inset of the free latissimus dorsi muscle flap (end-to-end anastomosis to the superficial temporal vessels), with its skin paddle. *Printed with permission; copyrights retained by Elise Lupon, MD, PhD.*
